# Optical excitation and external photoluminescence quantum efficiency of Eu^3+^ in GaN

**DOI:** 10.1038/srep05235

**Published:** 2014-06-10

**Authors:** W. D. A. M. de Boer, C. McGonigle, T. Gregorkiewicz, Y. Fujiwara, S. Tanabe, P. Stallinga

**Affiliations:** 1 Van der Waals-Zeeman Institute, University of Amsterdam, Science Park 904, 1098 XH Amsterdam, The Netherlands; 2 Division of Materials and Manufacturing Science, Graduate School of Engineering, Osaka University, Osaka, Japan; 3 Graduate School of Human and Environmental studies, Kyoto University, Kyoto, Japan; 4 FCT-DEEI, University of The Algarve, 8005-139 Faro, Portugal

## Abstract

We investigate photoluminescence of Eu-related emission in a GaN host consisting of thin layers grown by organometallic vapor-phase epitaxy. By comparing it with a reference sample of Eu-doped Y_2_O_3_, we find that the fraction of Eu^3+^ ions that can emit light upon optical excitation is of the order of 1%. We also measure the quantum yield of the Eu-related photoluminescence and find this to reach (~10%) and (~3%) under continuous wave and pulsed excitation, respectively.

Wide-gap semiconductors doped with rare-earth ions are of great interest for applications in light-emitting diodes (LEDs), because of their temperature insensitive, sharp and stable emission and an ease of current injection^1^,^2^. Blue and green LEDs based on galliumnitrides are already successfully commercialized. However, red emission from these materials is still lacking and monolithic full-color displays are at this moment impossible until this problem is solved. Large effort is thus spent on research of GaN materials that emit in the red. Among them, Eu-doped GaN (GaN:Eu) attracts special attention for its intense red emission around 622 nm, originating from the ^5^*D*_0_ → ^7^*F*_2_ transition of the Eu^3+^ ions^3^,^4^,^5^,^6^,^7^. In combination with Er^3+^ and Tm^3+^ doped GaN, GaN:Eu could be applied in monolithic full-color displays^8^. In fact, for displays with the full color gamut, a phosphor with an emission line around 610 nm is needed, and it is clear that only the Eu^3+^ ion can satisfy this requirement^9^. However, the light output power of the presently available GaN:Eu LEDs is still too low to compete with ‘conventional' red-light LEDs based on III-V materials. To overcome this, material characteristics of GaN:Eu layers need to be optimized. For that, we have to determine the limiting factor in the optical performance.

The main concerns are the optical accessibility – the percentage of Eu^3+^ ions which contribute to the emission – and the external quantum efficiency (of both electro- and PL). Past research has revealed that a variety of Eu-related emitting centers, as well as a variety of energy transfer routes exist^10^,^11^,^12^,^13^,^14^. These findings imply that the potential for improvement of emission is mainly to be found in material engineering towards optimization of the energy transfer between the GaN-host and Eu^3+^ ions^3^,^4^,^5^,^6^. We present here in this work the careful analysis of Eu luminescence in GaN and come to the conclusion that the problem lies in the low percentage of Eu that is optically active. We were able to draw this conclusion by comparing our GaN:Eu sample to a well-characterized standard reference sample of Y_2_O_3_:Eu. The europium in this reference sample, by being embedded in a non-active and insulating host matrix without any defects that can act as quenching centers, has an internal quantum efficiency of over 90% with, moreover, basically all Eu is participating in emission; the reference sample can thus be used as a way to calibrate the optical accessibility of europium in other matrices.

GaN:Eu material can be prepared in various ways. In particular, samples with high crystalline quality can be produced by organometallic vapor phase epitaxy (OMVPE, the de facto standard for GaN growth). For this technique, it has been shown that samples grown under atmospheric pressure feature significant enhancement of PL compared to those grown under low pressure, while the absolute Eu^3+^ concentration is smaller. Since the effective PL lifetime is not affected, this implies that the enhancement is due to a larger number of Eu^3+^ ions contributing to emission^3^,^4^. In this study we quantify this notion; we investigated the level of optical accessibility of Eu^3+^ and its temperature dependence in GaN layers grown by OMVPE and determined the fraction of Eu^3+^ dopants participating in emission to be in the order of only some percent. We also determined the external quantum yield (QY) of the Eu-related PL under different excitation conditions.

## Experimental

In this study, the GaN:Eu layers were grown by the OMVPE technique on a sapphire (0001) substrate. Specific details on the sample preparation procedure can be found in Ref. 4. The particular sample used in this study has the optically active GaN:Eu layer with a thickness of approximately 400 nm and nominal concentration of Eu^3+^ ions *N*_Eu_ = 3 × 10^19^ cm^−3^, as measured by secondary-ion mass-spectroscopy. The reference sample is a conventional industrial Y_2_O_3_ powder with a high concentration of Eu^3+^ ions, *N*_Eu_ = 1.16 × 10^21^ cm^−3^, compressed to a solid pellet to facilitate handling in optical experiments. Y_2_O_3_ is an insulating host typically used in rare-earth-based efficient phosphors; the wide band-gap character of this host makes that the Eu^3+^ ions can only be excited resonantly and can attain near 100% optical accessibility. Therefore, the chosen material can be suitably used as a reference, allowing for the determination of the percentage of Eu^3+^ ions contributing to the photo-luminescence, i.e., being optically accessible.

The PL experiments were conducted under pulsed and continuous wave (CW) excitations. For the former, a Nd:YAG-pumped optical parametric oscillator with a repetition rate of *f* = 100 Hz and a pulse duration of 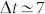 ns was used to obtain resonant excitation of the Eu in Y_2_O_3_. For GaN-host-mediated excitation, the 3^rd^ harmonic of the same laser pump was used, *λ*_exc_ = 355 nm. The CW GaN-mediated excitation was achieved with an Ar-ion laser operating at a wavelength of *λ*_exc_ = 364 nm. The spectral information was resolved by a multi-grating monochromator and detected by a CCD camera; time-resolved PL signals were registered with a water-cooled photo-multiplier tube with 1 μs resolution in the applied settings. The QY measurements were performed in both pulsed (resonant) and CW (host-mediated) excitation conditions, where for the latter a low-intensity xenon lamp was used in combination with a monochromator selecting the appropriate excitation wavelength, and recorded with a CCD camera. The sample was placed inside an integrating sphere for homogeneous distribution of the emitted and excitation light; The evaluation of the PL QY was done following the procedure of Refs. 15,16,17.

For the evaluation of the optical accessibility of Eu^3+^ in GaN, the integrated PL intensity was measured as a function of the applied photon fluence per pulse. The methodology is based on a comparison of the measured PL intensity with that of Eu^3+^ ions in the reference sample. In that way, the experimentally measured PL intensity can be directly related to a specific concentration of Eu^3+^ contributing to emission^18^,^19^. In this experimental approach, identical excitation and detection configurations for both samples were maintained in order to ensure constant alignment, independent of the excitation settings. A pinhole placed in front of the investigated sample defines the dimensions of the excitation spot, which, in combination with the exact measurement of the energy per laser pulse after the pinhole, determines the photon fluence value (the number of photons per cm^2^ per pulse, Φ).

## Results

**Figure 1:**
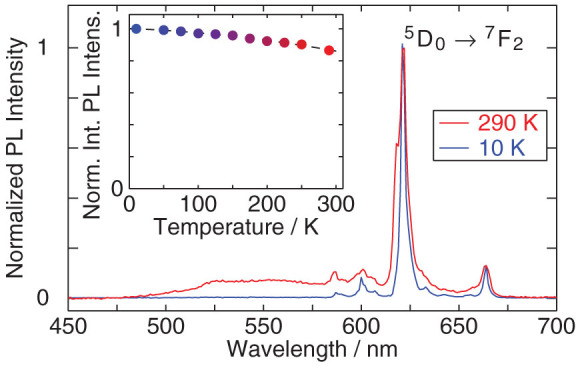
PL spectra of GaN:Eu measured under CW excitation at *T* = 10 K (blue line) and *T* = 290 K (red line). The inset shows the temperature dependence of the wavelength-integrated PL intensity.

For the initial optical characterization, the sample was measured upon CW band-to-band excitation, as done in previous studies on similar materials^3^,^4^,^5^,^6^. Figure 1 shows the normalized PL spectra of GaN:Eu measured at room temperature and at *T* = 10 K in red and blue, respectively. The peaks associated with the intra-4*f* shell transitions are visible^9^, with the main contribution arising from the ^5^*D*_0_ → ^7^*F*_2_ transition at 621.5 nm, as labeled in the figure. In addition to the Eu^3+^-related PL peaks, the spectrum measured at room temperature (red) shows a broad structure centered around *λ* = 575 nm and some 100s of nm wide, which decreases in intensity for lower temperatures. This feature appeared independently of the excitation photon flux – it has a constant relative amplitude – and can possibly be assigned to a recombination path involving defects and is not further discussed here. With the exception of this specific band, the PL spectral line positions of the investigated material are independent of temperature. The inset of Fig. 1 shows the temperature dependence of the PL intensity, obtained under a photon flux of *j*_exc_ = 9.4 × 10^18^ cm^−2^s^−1^. Saturation at low temperatures was seen, in agreement with previous reports^4^.

**Figure 2:**
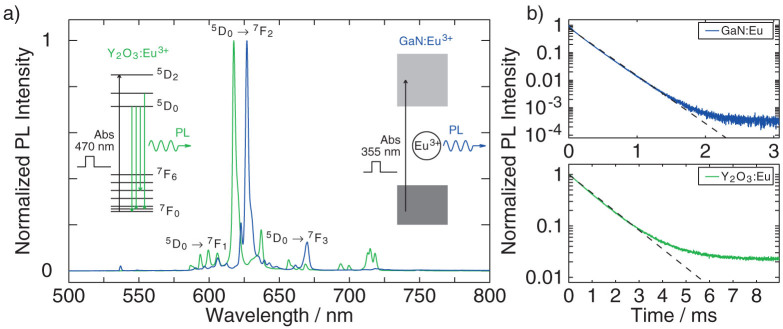
(a) PL spectra of GaN:Eu and Y_2_O_3_:Eu at room temperature under pulsed excitation, with the peaks labeled according to convention^9^ and consistent with measurements by Tallant et al.^21^. The insets show the respective excitation paths: the Eu^3+^-ions in GaN are excited by means of an indirect excitation mechanism, where photons are initially absorbed by the host, with the subsequent energy transfer to Eu^3+^ ions. In case of Y_2_O_3_:Eu, excitation is accomplished via direct resonant pumping of the ^7^*F*_0_ → ^5^*D*_2_ transition. (b) Decay profiles of PL from GaN:Eu (upper panel) and Y_2_O_3_:Eu (lower panel), measured at a wavelength of the maximum PL intensity, corresponding to recombination through the ^5^*D*_0_ → ^7^*F*_2_ transition.

The experiments for the evaluation of the percentage optical accessibility have been performed under pulsed excitation. The reference Y_2_O_3_:Eu sample was excited resonantly (*λ*_exc_ = 470 nm, coinciding with the ^7^*F*_0_ → ^5^*D*_2_ transition^9^,^20^,^21^), while the GaN:Eu sample was pumped with over-bandgap-energy photons. Figure 2(a) shows the normalized PL spectra of both GaN:Eu and Y_2_O_3_:Eu at room temperature in black and blue, respectively, with the corresponding excitation mechanisms schematically illustrated in the insets. In both samples, multiple peaks due to the intra-4*f* shell transitions are observed (labeled in the figure), with the whole emission band being clearly blue-shifted for Y_2_O_3_:Eu in comparison to GaN:Eu. The dominant PL feature in both cases can be associated with the ^5^*D*_0_ → ^7^*F*_2_ transition^9^, and is observed at *λ*_max_ = 612 nm for Y_2_O_3_:Eu and *λ*_max_ = 621.5 nm for GaN:Eu. Apart from the blue-shift of the main PL line, the influence of the host matrix can also be observed in the effective PL lifetime of the dominant peak, shown in Fig. 2(b). While in both materials a mono-exponential decay is measured – as indicated by dashed lines – the time constants differ by almost an order of magnitude, being *τ*_eff_ ≈ 250 μs (GaN, upper panel) and *τ*_eff_ ≈ 1.2 ms (Y_2_O_3_, lower panel).

**Figure 3:**
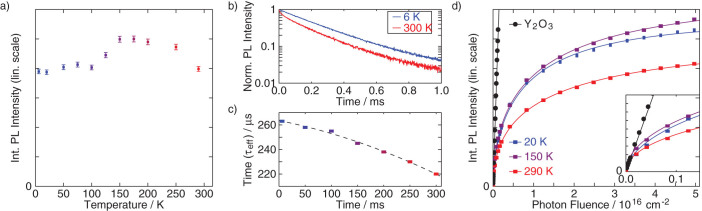
(a) Temperature dependence of the integrated PL of GaN:Eu for the temperature range of *T* = 6–300 K upon pulsed excitation with the photon fluence Φ = 4.6 × 10^16^ cm^−2^. (b) Decay profile of PL in GaN:Eu at *T* = 6 and *T* = 300 K. (c) Temperature dependence of the PL decay time of GaN:Eu. (d) Time-integrated PL intensity of GaN:Eu at *T* = 20 K, *T* = 150 K and *T* = 290 K and Y_2_O_3_:Eu at *T* = 290 K as a function of the applied photon fluence. The solid lines show linear (Y_2_O_3_) and stretched exponential (GaN) fits to the data. Parameters of the fits are given in Table 1.

The PL intensity measurements were conducted from room temperature cooling down. Figure 3(a) shows the temperature dependence of the integrated PL intensity at a photon fluence of Φ = 4.6 × 10^16^ cm^−2^. As can be seen, upon cooling, the PL intensity initially increases − down to *T* = 150 K – and then quenches somewhat. A similar dependence has been reported before for a Mg^2+^ co-doped GaN:Eu sample upon CW over-bandgap excitation^22^,^23^,^24^. Such behavior might be due to thermal activation of specific defect centers participating in excitation and de-excitation paths, which can then either enhance or quench the PL. At the same time, the lowering of temperature results in a moderate increase of PL effective lifetime from 220 μs to 265 μs, as shown in Figs. 3(b) and 3(c). This lack of correlation between PL intensity and lifetime temperature dependencies indicates that the effect of temperature is stronger on the excitation than on the recombination, as argued by Lee et al.^22^. The difference between the temperature dependence of the integrated PL intensity of GaN:Eu observed in our research and the research reported by Fujiwara et al. is most likely due to the difference between pulsed and CW excitation^3^,^4^,^5^,^6^. The differences are consistent with the concept of a variety of different Eu^3+^ centers, and site-selective excitation paths with individual thermal characteristics, as concluded in the past on basis of high-resolution PL and PL excitation investigations^10^,^11^,^12^,^13^,^14^.

Figure 3(d) shows the integrated PL spectra of Y_2_O_3_:Eu and GaN:Eu as a function of the applied photon fluence. The red, purple and blue squares correspond to the integrated PL intensity of GaN:Eu at *T* = 290 K, *T* = 150 K and *T* = 20 K, respectively. Initially, in the low photon fluence regime, a linear dependence can be observed for the sample, after which saturation sets in; a stretched exponential was fit to the data and shown as solid lines in the figure. It has to be noted that the PL spectrum remained unaltered within the investigated excitation flux range. The black circles depict the integrated PL intensity of the Y_2_O_3_:Eu reference sample measured at room temperature, and the solid black line is a linear fit to the data.

## Discussion

Based on this, an estimation of the optical accessibility of Eu^3+^ in GaN can be made by comparing the time-integrated PL intensity of the investigated sample with that of the Y_2_O_3_:Eu reference. The instantaneous PL intensities of both samples are proportional to 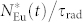, where 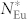 and *τ*_rad_ correspond to the density of excited Eu^3+^-ions and their radiative lifetimes, respectively. From here on, omitting the time (*t*) signifies the number at *t* = 0. Since the PL signals are integrated over time, the result will be proportional to 
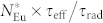
, where *τ*_eff_ is the effective PL lifetime, as measured in the experiment, and 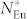 the initial number of excited Eu ions. (Note: The ratio of effective and radiative lifetimes is the internal quantum efficiency – number of photons created divided by the number of photons absorbed − IQE = *τ*_eff_/*τ*_rad_). To find the number of photons coming out of the sample and the external quantum efficiency (EQE), this value has to be multiplied by the extraction efficiency *η* that is determined by the refractive index of the material for that specific wavelength; (EQE = *η* × IQE; We ignore in this definition other effects such as photon reabsorption, as well as light-insertion efficiencies, the latter not entering the calculation anyway). Therefore, the total equation for the ratio of the number of photons emitted by GaN:Eu and Y_2_O_3_:Eu is given by 
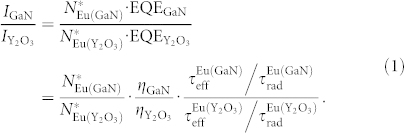
On basis of this, we can calculate the number of excited europium ions in our GaN sample, 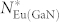, from the measured PL intensities *I*_GaN_ and 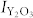. This goes as follows.

In the first step, we calculate the extraction efficiencies, knowing that these are determined by the refractive index of that particular material and wavelength. By Snell's law, light will not manage to come out at an angle greater than the critical angle that depends on the ratio of refractive indexes. For isotropic emission into air, it can be shown that the (double sided) extraction efficiency of a planar device is equal to 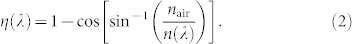
In our case, for GaN at *λ* = 621.5 nm, *n* = 2.39, and for Y_2_O_3_ at *λ* = 612 nm, *n* = 1.93^25^. With *n*_air_ assumed unity, the extraction coefficients can be calculated as: *η*_GaN_ = 0.091 and 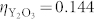.

The measured EQE of the Y_2_O_3_:Eu sample was 29.5% and this is much larger than the above implied maximum possible EQE of 14.4%. This shows that the above calculation for 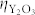 is incorrect, possibly due to a different geometry (a pellet rather than a planar device) and surface roughness. However, it does indicate that the internal quantum efficiency must be close to 100%. We continue, therefore, with a best guess of the extraction efficiency of 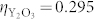 and an internal quantum efficiency of 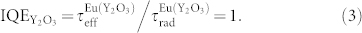
This introduces a substantial uncertainty in the following calculation. Continuing, the ratio of extraction efficiencies is then 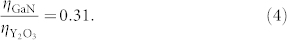
These values were used in our calculations.

Next, the effective lifetime of GaN:Eu PL, 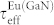, is measured via PL transients, as in Fig. 3(b). The radiative lifetime, 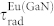, can be found from the radiative lifetime of Y_2_O_3_:Eu using Fermi's golden rule for spontaneous emissions that states that radiative lifetimes scale with the refractive index of the host material, *τ*_rad_ ∝ 1/*n*. With for Y_2_O_3_ the radiative and effective lifetimes equal (as discussed above) and the effective lifetime Y_2_O_3_, again, measured in transients, the radiative lifetime in GaN can be found as 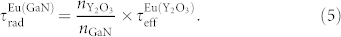
Finally, for this comparison we assume that all Eu^3+^-ions in Y_2_O_3_:Eu are equivalent and all contribute to the PL (in contrast to those in GaN:Eu). We can then calculate the number of excited europium atoms in Y_2_O_3_:Eu. Because the pulse duration in this experiment is much shorter than the effective lifetime *τ*_eff_ of Eu^3+^, it can be assumed that recombination does not take place during an excitation pulse and that 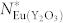 follows the equation^26^

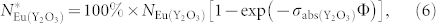
where 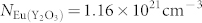 is the density of Eu-atoms, 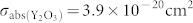 is the absorption cross-section of Eu^3+^ for the excitation wavelength *λ*_exc_ = 470 nm. It is based on a value given by Wakamatsu et al.^27^ for GaN scaled to Y_2_O_3_. As shown in Fig. 3(d) Y_2_O_3_:Eu was measured within the linear regime for low excitation density, where 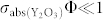. Therefore, the absorption for Y_2_O_3_:Eu is well described by the approximation 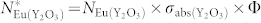. The PL intensity is proportional to this number and is thus a linear function of fluence, 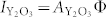. A fit to the data was made resulting in the value of the proportionality parameter 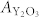 given in Table 1. The value given indicates that, for example, at a fluence of Φ = 10^16^/cm^2^ the Y_2_O_3_:Eu PL intensity is 8.254 times the saturation PL of GaN:Eu at 290 K.

For the PL of GaN:Eu, however, a stretched exponential behavior is observed, and the excitation of Eu atoms in GaN through absorption therefore follows the equation 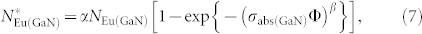
with *N*_Eu(GaN)_ = 3 × 10^19^ cm^−3^, and *α* the fraction (between 0 and 1) of ions that do take part in optical processes, i.e., the parameter we are trying to establish in this work. The PL is proportional to this number and follows a stretched exponential too. Fig. 3(d) shows that it fits the data quite well at all temperatures. The parameters of the fits are given in Table 1, with *A*_GaN_ the amplitude of the curves normalized to saturation PL at 290 K.

With this, the ratio of excited ions thus becomes 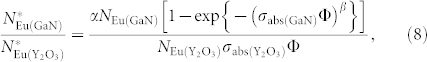
where *σ*_abs(GaN)_ and *β* are found from fitting a curve to the data. Note that, to get a rough idea, *σ*_abs(GaN)_ is basically the reciprocal fluence at which the PL intensity reaches 63% (1 − *e*) of its saturation value.

With the PL behavior known (*I*_GaN_ and 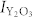, see Fig. 3(d)) – the geometric photon collection efficiencies are equal and thus cancel in the ratio, i.e., no integrating sphere needs to be used – the only remaining unknown in the starting equation (Eq. 1) is the fraction of active europium centers in GaN:Eu, *α*, which can thus be derived. As an example, for a fluence of Φ = 10^16^ cm^−2^, a ratio of 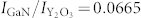 can be found from Fig. 3(d) or Table 1 for *T* = 290 K. When we substitute this into Eq. 1, with the parameters of Table 2 we get a value of 2.6%. In the same way, we find values of 2.9% and 3.3% for 20 K and 150 K, respectively.

While the particular experimental measurements leading to these numbers are quite accurate (PL intensity vs. photon fluence), the overall accuracy of the optical accessibility determination depends on the method itself and the assumptions which have been made. The errorbar on the final value is estimated to be about a factor 2, which is mainly due to the poor knowledge of the extraction efficiency as discussed before. Other factors that might influence it are the concentration on Eu^3+^ ions in the reference sample and the assumption that all of them contribute to PL. Finally, (multiple) internal reflections might play a role. Using Fresnel's equation (for perpendicular rays), light is reflected off the interfaces of air-GaN, GaN-sapphire, and sapphire-air. Using the refractive indexes of these materials (2.4, 1.77), we estimate that effectively 83% of the incident light is in the GaN layer under study. The absorption cross section found by us is then 17% under-estimated. This further increases the error bar. Yet, undoubtedly, a lot of europium ions are in the GaN host that never participate in any way in luminescence and this is the main conclusion of this work.

In addition, the PL QY of GaN:Eu was measured upon low flux CW (Xe lamp) and pulsed indirect excitation at *λ* = 355 nm. This method provides information on the efficiency of the energy transfer, as it gives directly the ratio of the number of emitted and absorbed photons, without consideration of the concentration of the available optically active centers. Under CW excitation, the PL QY was ≈3.2%, while under pulsed excitation it was found to be about a factor 2.5 higher, QY ≈ 8%. This difference is in agreement with the results observed in the PL spectra, since the defect-related emission shoulder, as revealed for CW excitation, is likely to lower the overall QY.

## Conclusions

The current work showed that the problem of low yield in GaN:Eu LEDs lies in the relatively low levels of optical accessibility of Eu dopants, in the order of a few percent. Here thus lies the opportunity for improvement, basically in material preparation.

The temperature-dependent percentage of optically active Eu^3+^ ions – the ‘optical accessibility' – has been determined for state-of-the-art GaN:Eu layers grown by OMVPE. The values between 2.6% and 3.3% have been found depending on the temperature (*T* = 20–300 K). This has been achieved by comparison of the integrated PL intensity of Eu-related emission in the investigated materials and a reference sample. Also the external quantum yield of the host-mediated Eu PL in GaN has been determined as QY ≈ 10% and QY ≈ 3%, for pulsed and CW band-to-band excitation modes, respectively.

## Author Contributions

Wd.B. and T.G. conceived the project and designed the experiments, Ch.Mc.G. and Wd.B. performed the experiments, Y.F. and S.T. supplied the GaN structure and the Y_2_O_3_ reference, Wd.B., Ch.Mc.G. and P.S. analyzed the data, Wd.B., P.S. and T.G. wrote the manuscript, T.G. facilitated and supervised the project. All the authors contributed to the analysis and data interpretation, and discussed the manuscript.

## Figures and Tables

**Table 1:** Parameter values found by fitting

Parameter	20 K	150 K	290 K
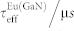	260	245	220
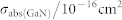	0.891	1.258	1.570
*β*	0.583	0.530	0.506
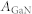	1.128	1.292	1
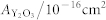			8.254
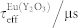			1200

**Table 2:** Parameter values used in this work

Parameter	value
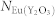	1.16 · 10^21^ cm^−3^
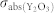	3.9 · 10^−20^ cm^2^
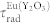	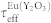
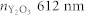	1.93
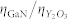	0.31
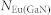	3 · 10^19^ cm^−3^
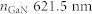	2.39
